# The Geographical Distribution of Human Cutaneous and Visceral *Leishmania* Species Identified by Molecular Methods in Iran: A Systematic Review With Meta-Analysis

**DOI:** 10.3389/fpubh.2021.661674

**Published:** 2021-06-25

**Authors:** Homa Hajjaran, Reza Saberi, Alireza Borjian, Mahdi Fakhar, Seyed Abdollah Hosseini, Sajjad Ghodrati, Mehdi Mohebali

**Affiliations:** ^1^Department of Medical Parasitology and Mycology, School of Public Health, Tehran University of Medical Sciences, Tehran, Iran; ^2^Toxoplasmosis Research Center, Communicable Diseases Institute, Iranian National Registry Center for Lophomoniasis and Toxoplasmosis, School of Medicine, Mazandaran University of Medical Sciences, Sari, Iran; ^3^Student Research Committee, Mazandaran University of Medical Sciences, Sari, Iran

**Keywords:** *Leishmania major*, *Leishmania tropica*, *Leishmania infantum*, DNA-based molecular method, human, Iran

## Abstract

Leishmaniasis is one of the most common vector-borne parasitic diseases in Iran. *Leishmania* species identification is necessary for epidemiological aspects, precise prognosis, control and treatment of the disease. We systematically searched all the studies, reports, and documentation related to species identification and geographical distribution of causative agents of cutaneous (CL), mucosal (ML), and visceral leishmaniasis (VL) using DNA-based molecular diagnostic techniques in Iran. International databases including PubMed, ScienceDirect, Embase, Google Scholar, Scopus, and Web of Science were systemically searched for English articles and Iran's databases including SID, IranMedex and Magiran were searched for Persian reports and articles. Searches were performed from 1999 to 2019 (20 years). The current review was conducted using the keywords: cutaneous leishmaniasis, visceral leishmaniasis, *Leishmania* species, Human, Molecular, PCR, and Iran. The study quality was evaluated using the NOS checklist. This meta-analysis procedure was accomplished using STATA, version 2.7.9. Of the 3,426 records identified in the initial search, 154 articles met inclusion criteria and qualified for the systematic review and meta-analysis. In subgroup analysis, the pooled frequency of causative agents of CL isolates was 67.3% (95% CI: 59.51–74.67%) for *L. major* and 32.1% (95% CI: 24.72–39.87%) for *L. tropica*. In addition, the pooled frequency of causative agents of VL isolates was 97.1% (95% CI: 94.6–98.8%) for *L. infantum* and 2.9% (95% CI: 1.12–5.37%) for *L. tropica*. The findings of this study showed that the main causative agents of CL and VL in Iran are *L. major* and *L. infantum*, respectively. Moreover, kinetoplast DNA (kDNA) and internal transcriber spacer (ITS) were the most used markers for identifying *Leishmania* species. The current study provides valuable data to encourage and direct researchers as well as public health managers in the comprehensive leishmaniasis control and prevention planning in Iran.

## Introduction

Leishmaniasis is a neglected tropical disease (NTD) caused by the *Leishmania* parasites, which are transmitted by the bite of sand flies (1). There are four clinical forms of the disease: cutaneous leishmaniasis (CL), visceral leishmaniasis (VL), and mucocutaneous leishmaniasis (MCL) and mucosal leishmaniasis (ML) (2). Despite universal scientific community efforts to reduce cases of human leishmaniasis, numerous cases of such devastating disease are still reported worldwide (3). The disease currently affects 12 million people with 350 million people are living in regions with a high risk of infection. World Health Organization (WHO) estimates the annual global incidence of 0.7–1.2 million cases of CL and 0.1–0.4 million cases of VL (4). At present, the majority (about 90%) of CL cases occur in eight countries mainly including Asian and South American countries (4). Moreover, more than 90% of global cases of VL had been reported from seven countries mainly including African and South American countries (4, 5). In Iran, CL is the most common form of the disease and recent reports estimates >20,000 annual cases (6), but VL has been reported sporadically, with about 100–300 new serologically positive cases of VL reported annually (7).

Species discrimination is important, because of differences among the *Leishmania* species in levels of virulence and responses to the various chemical drugs (8, 9). As a result, distinguishing *Leishmania* spp. is critical for accurate diagnosis and appropriate treatment (9). Morphological identification of *Leishmania* species is not possible, but a variety of DNA-based molecular diagnostic techniques, including restriction fragment length polymorphism (RFLP), nested-PCR methods as well as high-resolution melting analysis PCR (HRM-PCR) have been reported for identification of *Leishmania* on different taxonomical levels (genus and species) (10). According to our literature review, several target markers were used to identify *Leishmania* species, including minicircle kinetoplastic DNA, heat shock protein 70 gene, N-acetylglucosamine-1-phosphate transferase (nagt) gene, and internal transcription spacer (ITS1 & 2).

There are several studies regarding the identification of *Leishmania* species causing CL and VL in Iran. The aim of this systematic review and meta-analysis was therefore to define the geographical distribution of *Leishmania* spp. among human populations as well as exploring molecular markers used for identifying *Leishmania* spp. in this population throughout two decades ago (1999–2019) in Iran.

## Methods

### Search Approach

This systematic study was achieved according to the Preferred Reporting Items for Systematic Reviews and Meta-Analysis (PRISMA) (11). The present study was carried out to estimate the species identification and geographical distribution of causative agents of CL and VL cases in Iran. A search in literature was carried out via the nine English and Persian databases, including PubMed, Embase, Google Scholar, Science Direct, Scopus, Web of Science and SID, IranMedex and Magiran up to Sep 2019, respectively. The current review was conducted by the Medical Subject Headings (MeSH) terms including: “Cutaneous leishmaniasis”, “Visceral leishmaniasis,” “*Leishmania”*, “Species”, “Human,” “Molecular”, “PCR”, and “Iran”, alone or combined together with “OR” or/and “AND” operators.

### Paper Screening

Initially, the titles and abstracts of searched articles were screened for eligibility by two authors independently, and those that did not describe identification of *Leishmania* species were removed. Data on the identification of *Leishmania* spp., were extracted from studies according to the following including criteria: (a) peer-reviewed original research, (b) papers studies that surveyed identification of *Leishmania* species using various polymerase chain reaction techniques, (c) studies published in English or Persian during 1999–2019 and (d) full-text articles were available. Additionally, the exclusion criteria were as follows; (a) duplicated data, (b) review studies, and (c) studies on animal reservoirs.

### Data Extraction

Out of the retrieved papers, 154 papers were eligible for inclusion in this study. Required data were collected based on the first author, publication year, province, total sample, positive number, *Leishmania* spp., types of clinical manifestation, diagnostic methods, marker genetic used and quality assessment. Three independent authors extracted the above details carefully.

### Quality Assessment

In the current study, the Newcastle-Ottawa Scale was used to evaluate the quality of studies. NOS score ranged from 0 and 7 [low quality, (1 and 2), moderate quality, (3–5), and high quality (6 and 7)] (12).

### Statistical Analysis

This meta-analysis was completed using STATA software, as comprehensive meta-analysis software (http://statsdirect.com). The heterogeneity index was assessed using standard Cochran's Q- and I-squared statistics, with the random effects estimate they imply. Egger's test was used to assess potential publication bias. A *p* < 0.05 (≤0.05) is statistically significant.

## Results

### Characteristics and Quality of the Included Studies

**Figure 1:**
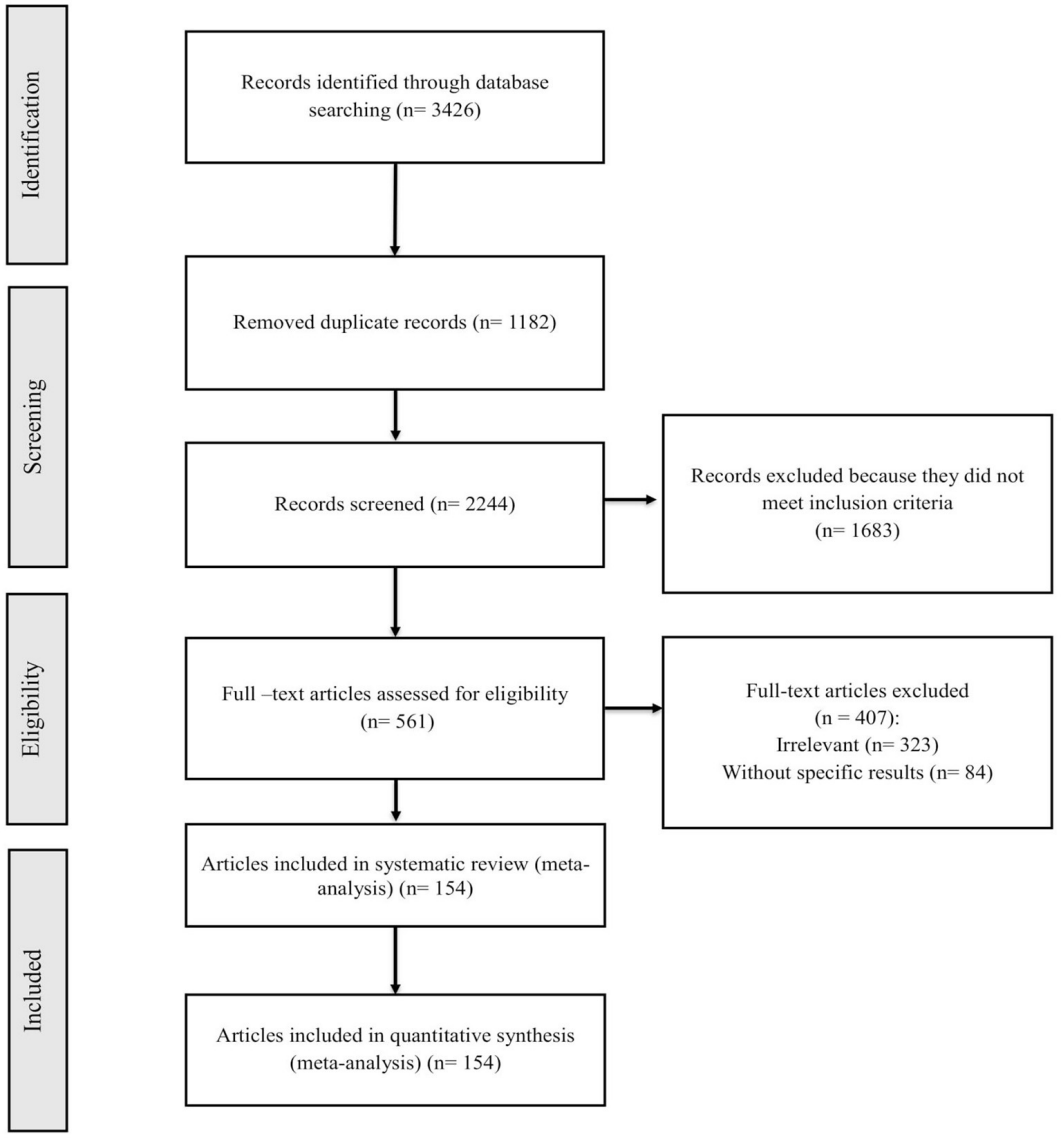
PRISMA flow diagram describing summaries of included/excluded studies.

Records retrieved in the mentioned electronic databases based on preparatory search strategies of nine databases yielded 3,426 papers; after removal of duplication papers, 2,244 papers were extracted. In the next step, using the abstract screening based on the inclusion/exclusion criteria, 1,683 other articles were excluded. Following that, 561 full-text articles were screened, of which 154 were found to be eligible for systematic review and meta-analysis. Figure 1 summarizes the flow chart presenting the study design process. The baseline characteristics of all included studies are tabulated in Tables 1, 2.

**Table 1:** Baseline characteristics of the *Leishmania* species identification from CL cases in the systematic review and meta-analysis from 1999 to 2019.

**References**	**Years**	**Province**	**Number samples**	**Species**		**PCR type**	**Used genetic marker**	**NOS score**
				***L. major***	***L. tropica***			
Alimohammadian et al. (13)	1999	Isfahan	8	8	0	PCR	kDNA	5
Motazedian et al. (14)	2002	Iran (Several province)^a^	78	42	36	RAPD-PCR	Random Primers	3
Tashakori et al. (15)	2003	Iran (Several province)^b^	67	45	22	PCR	kDNA	4
Hajjaran et al. (16)	2004	Razavi Khorasan	87	5	82	RAPD-PCR	Random Primers	4
Motazedian et al. (17)	2004	Fars	47	27	20	Nested PCR	kDNA	4
Hadighi et al. (18)	2006	Razavi Khorasan	31	3	28	PCR	PTR1	3
Tashakori et al. (19)	2006	Iran (Several province)^c^	24	24	0	RFLP	ITS_1&2	5
Akhavan et al. (20)	2007	Kerman	2	2	0	RAPD-PCR	Random primers	3
Maraghi et al. (21)	2007	Khuzestan	100	90	10	Nested PCR	kDNA	4
Kazemi rad et al. (22)	2008	Tehran	31	14	17	RFLP	ITS_1	6
Shahbazi et al. (23)	2008	Razavi Khorasan	86	3	83	RFLP	ITS_1	4
Fazaeli et al. (24)	2008	Sistan and Baluchestan	51	51	0	PCR	kDNA	4
Mohajery et al. (25)	2008	Razavi Khorasan	57	0	57	RAPD-PCR	Random primers	4
Alimoradi et al. (26)	2009	Kermanshah	20	17	3	RAPD-PCR	Random primers	3
Rahbarian et al. (27)	2009	Golestan	46	46	0	PCR	ITS_1&2	5
Razmjou et al. (28)	2009	Fars	27	27	0	Nested PCR	kDNA	6
Emami et al. (29)	2009	Isfahan	28	28	0	RAPD-PCR	Random primers	4
Fazaeli et al. (30)	2009	Sistan and Baluchestan	41	41	0	PCR	kDNA	4
Khalili et al. (31)	2009	Kerman	55	0	55	RFLP	ITS_1	5
Khalili et al. (31)	2009	Fars	28	1	27	RFLP	ITS_1	4
Pirstani et al. (32)	2009	Razavi Khorasan	54	17	37	RFLP	ITS_1	3
Sharifi et al. (33)	2010	Kerman	9	0	9	PCR	kDNA	4
Doudi et al. (34)	2010	Isfahan	209	205	4	RFLP	ITS_1	3
Doudi et al. (34)	2010	Kerman	122	50	72	RFLP	ITS_1	3
Saki et al. (35)	2010	Khuzestan	60	58	2	RFLP	kDNA	5
Pourmohammadi et al. (36)	2010	Fars	204	196	8	PCR	kDNA	4
Mahmoodi et al. (37)	2010	Razavi Khorasan	21	2	19	PCR	kDNA	4
Mesgarian et al. (38)	2010	Golestan	46	46	0	PCR	ITS_1&2	5
Mohajery et al. (39)	2010	Razavi Khorasan	86	54	32	PCR	kDNA	4
Fakhar et al. (40)	2010	Fars	35	35	0	PCR	kDNA	4
Hamzavi et al. (41)	2010	Kermanshah	7	7	0	RAPD-PCR	RP	3
Ghasemian et al. (42)	2011	Khuzestan	100	97	3	Nested PCR	kDNA	4
Farahmand et al. (43)	2011	Tehran	50	32	18	PCR	kDNA	4
Hajjaran et al. (44)	2011	Tehran	51	32	19	RFLP	ITS_1	7
Sharifi et al. (45)	2011	Kerman	26	0	26	PCR	kDNA	6
Sharifi et al. (46)	2011	Kerman	66	0	66	PCR	kDNA	6
Khademvatan et al. (47)	2011	Khuzestan	95	90	5	RT-PCR	kDNA	5
Azani et al. (48)	2011	Semnan	25	25	0	RFLP	ITS_1	3
Pour et al. (49)	2011	Kerman	51	0	51	Nested PCR	kDNA	4
Azani et al. (50)	2011	Semnan	57	57	0	Nested PCR	kDNA	4
Poursmaelian et al. (51)	2011	Kerman	188	0	188	Nested PCR	kDNA	5
Azizi et al. (52)	2012	Hormozgan	18	18	0	Nested PCR	kDNA	4
Khosravi et al. (53)	2012	Isfahan	79	75	4	RT-PCR	Tryparedoxin reroxidase	3
Sharifi et al. (54)	2012	Kerman	203	9	194	Nested PCR	kDNA	4
Mahmoudzadeh et al. (55)	2012	Iran (Several province)^d^	341	283	58	PCR	kDNA	3
Shiee et al. (56)	2012	Isfahan	63	5	58	RFLP	ITS_1	4
Hashemi et al. (57)	2012	Isfahan	50	46	4	RFLP	ITS_1	4
Mirzaei et al. (58)	2012	Kerman	26	0	26	Nested PCR	kDNA	5
Mohammadi et al. (59)	2012	Isfahan	60	60	0	RFLP	ITS_1	4
Saghafipour et al. (60)	2012	Qom	15	15	0	RFLP	ITS_1	3
Baghaei et al. (61)	2012	Fars	32	31	1	RFLP	ITS_1	4
Baghaei et al. (62)	2012	Golestan	90	90	0	RFLP	ITS_1	4
Kazemi rad et al. (63)	2013	Razavi Khorasan	2	0	2	RFLP	ITS_1	3
Akhundi et al. (64)	2013	Fars	42	39	3	RFLP	ITS_1	4
Sharif maraghi et al. (65)	2013	Khuzestan	146	138	8	Nested PCR	kDNA	4
Kheirandish et al. (66)	2013	Lorestan	178	49	129	PCR	ITS_1	4
Yaghoobi Ershadi et al. (67)	2013	Bushehr	8	2	6	Nested PCR	ITS_1	5
Kheirandish et al. (68)	2013	Lorestan	62	17	45	PCR	ITS_1	4
Saadabadi et al. (69)	2013	Razavi Khorasan	22	0	22	RAPD-PCR	RP	3
Hajjaran et al. (70)	2013	Iran	114	75	39	RFLP	ITS_1	5
Oryan et al. (71)	2013	Fars	98	97	1	Nested PCR	kDNA	4
Aflatoonian et al. (72)	2013	Kerman	66	0	66	PCR	kDNA	4
Taghizadeh et al. (73)	2013	Isfahan	123	111	12	PCR	kDNA	5
Badrizadeh et al. (74)	2013	East Azerbaijan	12	12	0	Nested PCR	kDNA	4
Hoseini Farash et al. (75)	2013	Razavi Khorasan	136	0	136	PCR	kDNA	6
Mohammadi et al. (76)	2013	Tehran	255	147	108	PCR	ITS_2	5
Beiranvand et al. (77)	2013	Lorestan	52	50	2	Nested PCR	kDNA	5
Karamian et al. (78)	2013	South Khorasan	80	8	72	RFLP	ITS_1	3
Pagheh et al. (79)	2013	Golestan	50	50	0	PCR	kDNA	4
Mirzaie et al. (80)	2013	Yazd	102	50	52	RFLP	ITS_1	5
Spotin et al. (81)	2014	Khuzestan	99	90	9	RFLP	ITS_1,Cyt b,rDNA	6
Tolouei et al. (82)	2014	Isfahan	28	28	0	PCR	ITS_1	5
Shirian et al. (83)	2014	Fars	98	97	1	Nested PCR	kDNA	4
Salehi et al. (84)	2014	Razavi Khorasan	35	4	31	PCR	kDNA	4
Hajjaran et al. (9)	2014	Iran (Several province)e	77	36	41	RFLP	NAGT	6
Eslami et al. (85)	2014	Yazd	102	50	52	RFLP	ITS_1	3
Arjmand et al. (86)	2014	Isfahan	50	50	0	Nested PCR	ITS_1	4
Hassanpour et al. (87)	2014	Razavi Khorasan	86	54	32	PCR	kDNA	4
Ghatee et al. (88)	2014	Fars	31	22	9	PCR	ITS_1	4
Ghatee et al. (88)	2014	Kerman	119	0	119	PCR	ITS_1	4
Bordbar et al. (89)	2014	Golestan	123	123	0	RFLP	ITS_1&2	5
Moravvej et al. (90)	2014	Tehran	8	8	0	PCR	kDNA	4
Karimian Shirazi et al. (91)	2014	Razavi Khorasan	100	6	94	Semi-nested	kDNA	4
Abdolmajid et al. (92)	2015	Razavi Khorasan	66	20	46	PCR	kDNA	5
Shamsian et al. (93)	2015	Razavi Khorasan	64	52	12	PCR	kDNA	4
Spotin et al. (94)	2015	Khuzestan	97	97	0	RFLP	ITS_1,Cyt b	5
Mohebali et al. (95)	2015	Bushehr	21	14	7	RFLP	ITS_1	7
Doroodgar et al. (96)	2015	Isfahan	14	10	4	RAPD-PCR	RP	4
Kolivand et al. (97)	2015	Tehran	15	15	0	PCR	kDNA	3
Gholami et al. (98)	2015	Ilam	50	50	0	RFLP	ITS_1	4
Hezari et al. (99)	2016	Golestan	38	38	0	RFLP	ITS_1	5
Haddad et al. (100)	2016	Ilam	92	92	0	Nested PCR	kDNA	4
Naseri et al. (101)	2016	Razavi Khorasan	60	7	53	PCR	kDNA	4
Ghasemloo et al. (102)	2016	Isfahan	70	10	60	RFLP	ITS_1	4
Rasti et al. (103)	2016	Isfahan	96	26	70	Nested PCR	kDNA	4
Mirahmadi et al. (104)	2016	Sistan and Baluchestan	64	11	53	RFLP	Hsp70	5
Dabirzadeh et al. (105)	2016	Sistan and Baluchestan	19	19	0	RFLP	ITS_1	6
Sharifi rad et al. (106)	2016	Sistan and Baluchestan	35	35	0	PPIP-PCR	kDNA	4
Sarkari et al. (107)	2016	Fars	77	57	20	RFLP	NAGT	5
Abedi-Astaneh et al. (108)	2016	Qom	9	9	0	RFLP	ITS_1	6
Hajjaran et al. (109)	2016	Tehran	43	24	19	RFLP	ITS_1	5
Izadi et al. (110)	2016	Fars	54	49	5	Nested PCR	kDNA	4
Izadi et al. (110)	2016	Isfahan	25	25	0	Nested PCR	kDNA	4
Karamian et al. (111)	2016	South Khorasan	60	5	55	RFLP	ITS_1	4
Mohammadpour et al. (112)	2016	Fars	6	4	2	PCR	kDNA	4
Soltan et al. (113)	2016	Khuzestan	97	97	0	RFLP	ITS_1, Cyt b, rDNA	3
Pazoki Ghohe et al. (114)	2016	Tehran	57	57	0	PCR	kDNA	4
Rezai et al. (115)	2017	Razavi Khorasan	84	16	68	PCR	kDNA	5
Kermanjani et al. (116)	2017	Ilam	61	61	0	RFLP	ITS_1	5
Mohammadiha et al. (117)	2017	Razavi Khorasan	94	33	61	RFLP	ITS_1	3
Nemati et al. (118)	2017	Iran (Several province)f	24	15	9	RFLP	Hsp70	5
Motalleb et al. (119)	2017	Sistan and Baluchestan	100	53	47	RFLP	Cyt b	4
Esmaeili Rastaghi et al. (120)	2017	Golestan	87	85	2	RFLP	ITS_1	4
Esmaeili Rastaghi et al. (120)	2017	Khuzestan	87	87	0	RFLP	ITS_1	4
Esmaeili Rastaghi et al. (120)	2017	Yazd	52	48	4	RFLP	ITS_1	4
Behravan et al. (121)	2017	Tehran	44	37	7	RFLP	ITS_1	4
Mohammadpour et al. (122)	2017	Fars	6	3	3	PCR	kDNA	3
Saghafipour et al. (123)	2017	Qom	45	45	0	RFLP	ITS_1	4
Fata et al. (124)	2017	Razavi Khorasan	85	63	22	PCR	kDNA	6
Akia et al. (125)	2017	Kermanshah	47	40	7	RFLP	ITS_1	4
Mirahmadi et al. (126)	2018	Sistan and Baluchestan	98	53	45	RFLP	Cyt b	5
Namazi et al. (127)	2018	Razavi Khorasan	153	153	0	Nested PCR	kDNA	4
Teimouri et al. (128)	2018	Iran	108	48	60	RFLP	ITS_1	4
Zahirnia et al. (129)	2018	Yazd	52	48	4	RFLP	ITS_1	5
Ghatee et al. (130)	2018	Kerman	26	0	26	RFLP	kDNA	4
Ghatee et al. (130)	2018	Fars	13	0	13	RFLP	kDNA	4
Saberi et al. (8)	2018	Ilam	62	62	0	RFLP	NAGT	4
Mousavi et al. (131)	2018	Ilam	200	200	0	PCR	kDNA	4
Ramezany et al. (132)	2018	Kerman	174	20	154	PCR	ITS_1	4
Askari et al. (133)	2018	Ilam	160	160	0	RFLP	ITS_1	5
Mirzaei et al. (134)	2018	Ilam	23	15	8	PCR	kDNA	5
Fata et al. (135)	2018	Razavi Khorasan	42	29	13	PCR	kDNA	6
Gholamian et al. (136)	2018	Yazd	88	88	0	Nested PCR	kDNA	4
Mohammadiha et al. (137)	2018	Iran	654	478	176	RFLP	ITS_1&2	5
Mirzapour et al. (138)	2019	Lorestan	100	16	84	RFLP	ITS_1	6
Razavinasab et al. (139)	2019	Kerman	50	0	50	HRM	7SL RNA	5
Mohammadpour et al. (140)	2019	Fars	100	86	14	Nested PCR	kDNA, Cyt b	5
Barazesh et al. (141)	2019	Fars	66	60	6	PCR	kDNA	4
Ghobakhloo et al. (142)	2019	Fars	161	161	0	PCR	GAPDH	4
Mirahmadi et al. (143)	2019	Sistan and Baluchestan	111	68	43	RFLP	kDNA	5
Ziaei Hezarjaribi et al. (144)	2019	Kerman	40	0	40	PCR	kDNA	4
Zarezadeh et al. (145)	2019	Sistan and Baluchestan	82	36	46	RFLP	ITS_1, rDNA	5

*(Several province) ^a^Fars (n = 32), Kerman (n = 20), Tehran (n = 8), Isfahan (n = 7), Khuzestan (n = 4), Golestan (n = 2), and Yazd (n = 2)*.

*(Several province) ^b^Isfahan (n = 40), Ilam (n = 9), Razavi Khorasan (n = 7), Semnan (n = 6), and Khuzestan (n = 5)*.

*(Several province) ^c^Isfahan (n = 11), Khuzestan (n = 6), Semnan (n = 4), Ilam (n = 2), and Tehran (n = 1)*.

*(Several province) ^d^Isfahan (n = 59), Hormozgan (n = 47), Semnan (n = 43), Ilam (n = 42), Razavi Khorasan (n = 41), Fars (n = 30), Kerman (n = 21), Khuzestan (n = 21), Yazd (n = 20) Golestan (n = 13), and North Khorasan (n = 4)*.

*(Several province) ^e^Isfahan (n = 40), Razavi Khorasan (n = 15), Kermanshah (n = 11), Kerman (n = 9), Khuzestan (n = 1), Tehran (n = 1)*.

*(Several province) ^f^Isfahan (n = 4), Razavi Khorasan (n = 4), Fars (n = 4), Ilam (n = 3), Kerman (n = 2), Golestan (n = 2), Tehran (n = 1), Kermanshah (n = 1), Ardabil (n = 1), Hormozgan (n = 1), and Sistan and Baluchestan (n = 1)*.

**Table 2:** Baseline characteristics of the *Leishmania* species identification from the VL cases in the systematic review and meta-analysis from 2005 to 2019.

**References**	**Year**	**Province**	**Positive samples**	**Species**		**PCR type**	**Used genetic marker**	**NOS score**
				***L. infantum***	***L. tropica***			
Sarkari et al. (146)	2005	Kohgiluyeh and Boyer Ahmad	6	6	0	Semi Nested	kDNA	3
Alborzi et al. (147)	2006	Fars	64	63	1	PCR	kDNA	3
Motazedian et al. (148)	2008	Fars	29	29	0	PCR	kDNA	4
Alborzi et al. (149)	2008	Fars	95	95	0	PCR	kDNA	5
Fayzi et al. (150)	2009	East Azerbaijan	14	14	0	PCR	kDNA	4
Fakhar et al. (151)	2011	Kermanshah	9	9	0	PCR	kDNA	3
Fakhar et al. (152)	2011	Fars	16	16	0	PCR	kDNA	3
Hajjaran et al. (70)	2013	Iran	28	26	2	RFLP	ITS_1	5
Mohammadiha et al. (153)	2013	Ardabil	77	77	0	Real-time PCR	kDNA	4
Fakhar et al. (154)	2014	Golestan	13	13	0	PCR	kDNA	6
Hosseininasab et al. (155)	2014	Kerman	10	9	1	Nested PCR	kDNA	4
Ghasemian et al. (156)	2016	Tehran	45	45	0	Nested PCR—RT PCR	kDNA	4
Sarkari et al. (157)	2016	Fars	1	0	1	RFLP	NAGT	4
Hajjaran et al. (109)	2016	Tehran	7	4	3	Semi Nested RT PCR	NAGT, ITS_1	5
Asfaram et al. (158)	2017	Ardabil	16	16	0	PCR	kDNA	5
Asfaram et al. (159)	2017	Golestan	6	6	0	PCR	kDNA	4
Asfaram et al. (159)	2017	Mazandaran	1	1	0	PCR	kDNA	4
Dalimi et al. (160)	2018	Ardabil	14	14	0	RFLP	ITS_1	6
Dalimi et al. (160)	2018	Fars	9	9	0	RFLP	ITS_1	6
Dalimi et al. (160)	2018	Bushehr	3	3	0	RFLP	ITS_1	6
Dalimi et al. (160)	2018	East Azerbaijan	2	2	0	RFLP	ITS_1	6
Dalimi et al. (160)	2018	Tehran	2	2	0	RFLP	ITS_1	6
Masoori et al. (161)	2018	Lorestan	16	16	0	PCR	kDNA	5
Layegh Gigloo et al. (162)	2018	Fars	8	8	0	PCR	ITS_2	4
Rezaei et al. (163)	2018	Fars	8	8	0	PCR	ITS_2	5
Mirzaei et al. (134)	2018	Ilam	14	14	0	PCR	kDNA	5
Ghatee et al. (164)	2018	Kohgiluyeh and Boyer Ahmad	29	25	4	RFLP	ITS_1	4

### Results of the Meta-Analysis

**Figure 2:**
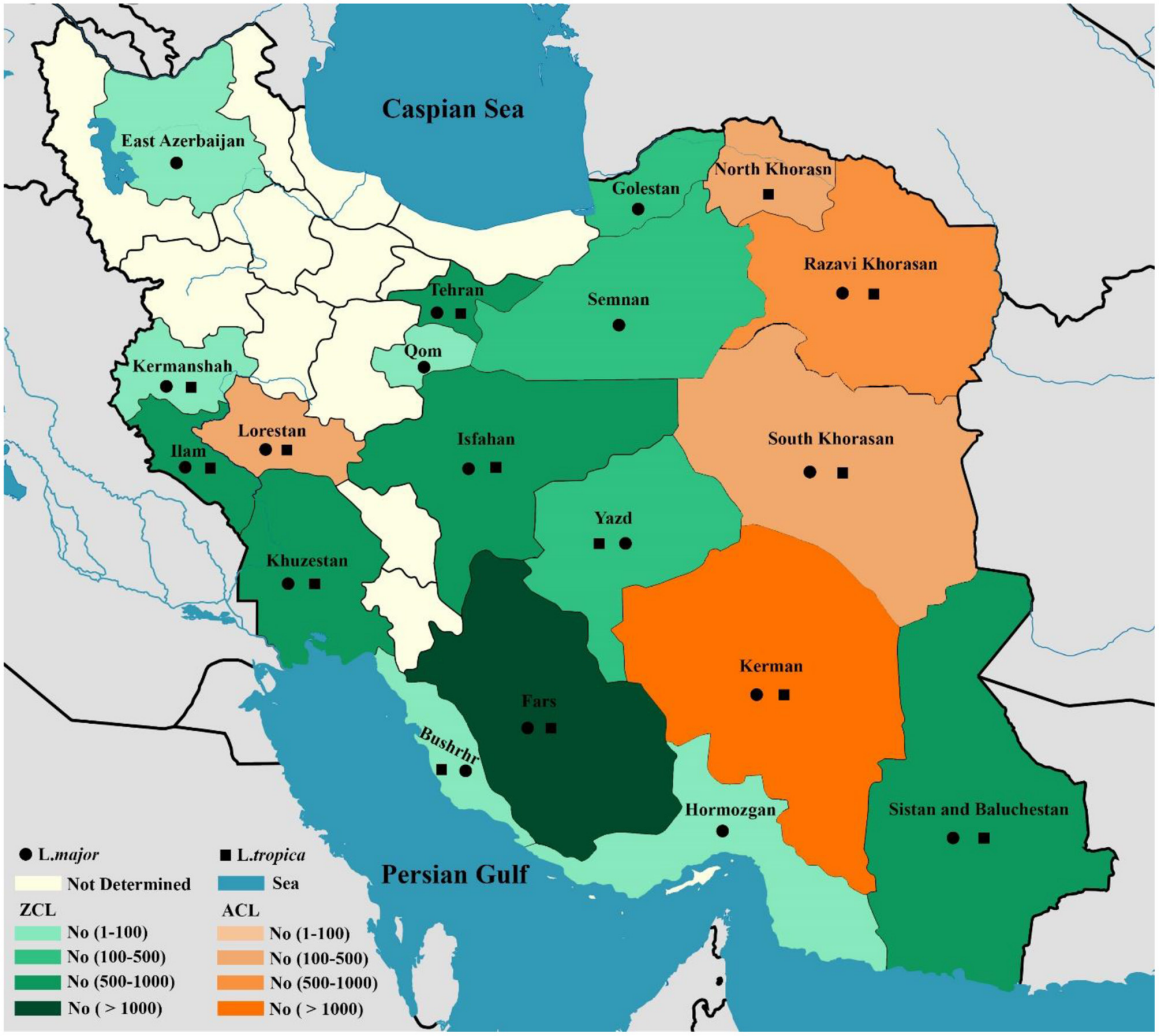
Map of distribution of *Leishmania* spp. causing ZCL and ACL using molecular methods in different geographical areas of Iran.

**Figure 3:**
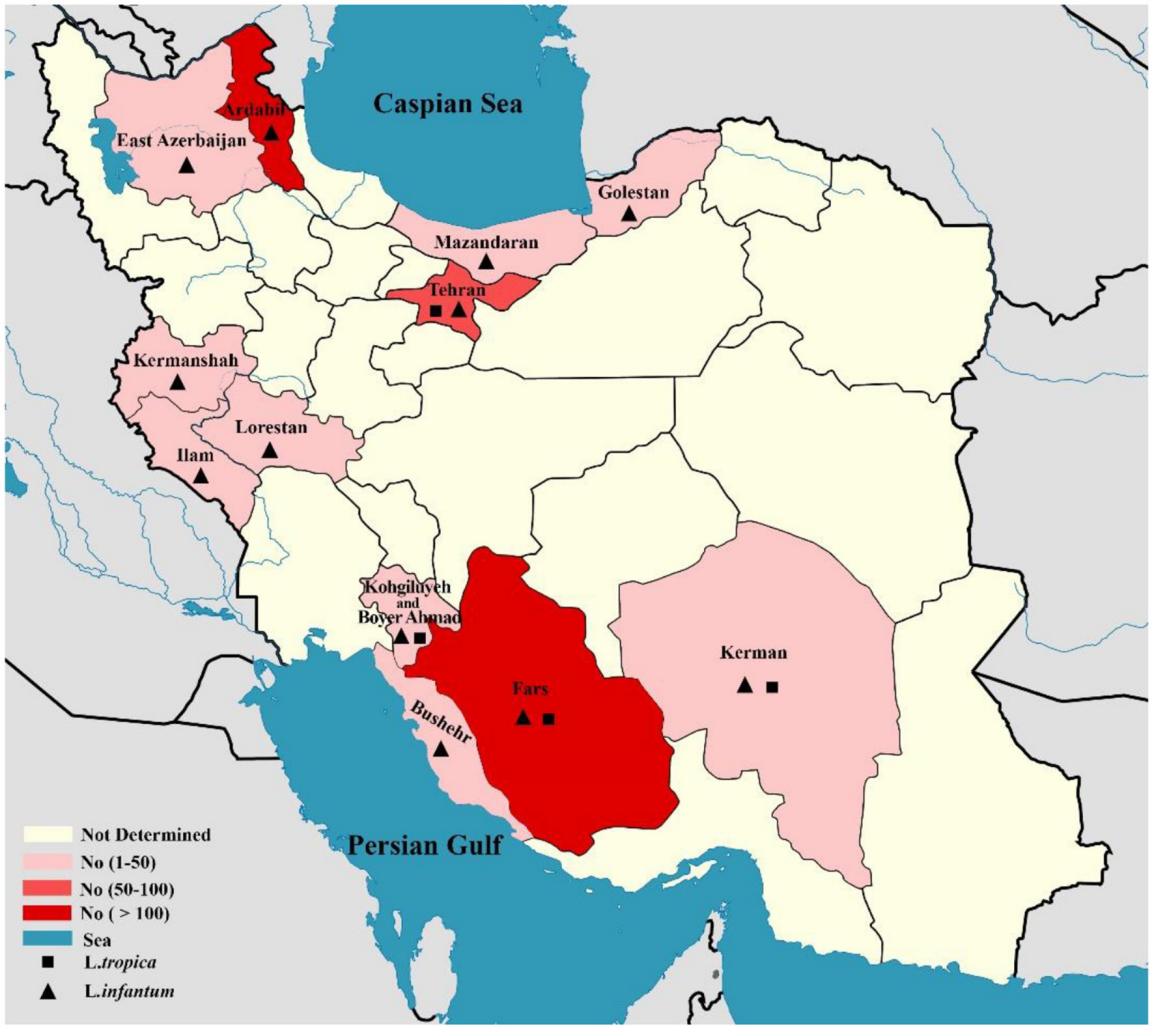
Map of distribution of *Leishmania* spp. causing of visceral leishmaniasis using molecular methods in different geographical areas of Iran.

In total, 10,586 CL isolates were identified, with two causatives of ZCL (L. major, *n* = 6,714) and ACL (*L. tropica, n* = 3,872) being reported in 19 provinces across Iran (Fars, Khuzestan, Isfahan, Golestan, Ilam, Razavi Khorasan, Kerman, Sistan & Balochistan, Tehran, Yazd, Hormozgan, Semnan. Most of the *L. major* isolates belonged to Fars (*n* = 992), Khuzestan (*n* = 844), and Isfahan (*n* = 687) provinces in the southern and central regions of Iran. In addition, the majority of *L.tropica* isolates belonged to Kerman (*n* = 1,142), Razavi Khorasan (*n* = 949) in the east, and Lorestan (*n* = 260) in the west (Figure 2). In contrast, 542 isolates for VL cases were determined in 11 provinces (Fars, Ardabil, Tehran, Kohgiluyeh and Boyer-Ahmad, Golestan, Ilam, Lorestan, East-Azerbaijan, Boushehr, Kerman, and Mazandaran), with the majority of VL isolates belonging to Fars (*n* = 230) in southwestern Iran and Ardabil (*n* = 107) in northwestern Iran (Figure 3).

According to the literature review, nine genetic markers (kinetoplast DNA, internal transcribed spacer region-1 and 2, cytochrome b, heat shock protein 70, N -acetylglucosamine-1-phosphate transferase, pteridine reductase 1, tryparedoxin peroxidase, Glyceraldehyde-3-Phosphate Dehydrogenase (GAPDH) and 7SL RNA) were used for identification *Leishmania* species that the most species were identified with kinetoplast DNA (*n* = 5,592) and ITS markers (*n* = 4,544). It should be noted that some species of *Leishmania* were identified by Random amplified polymorphic deoxyribonucleic acid analysis by PCR (RAPD-PCR) method using random primers. The used molecular methods of *Leishmania* species identification in most studies were nested PCR and PCR-RFLP.

In subgroup analysis, the pooled frequency of causative agents of CL isolates was 67.3% (95% CI: 59.51–74.67%) for *L. major* and 32.1% (95% CI: 24.72–39.87%) for *L. tropica* (Table 3 and Supplementary Figures 1, 2). Also of note, the 14 and four isolates were identified as *L. infantum* and *L. turanica* as causative agents of CL and ZCL cases, respectively (14, 22, 36, 47, 74, 89, 120, 122).

**Table 3:** The pooled frequency, heterogeneity, and publication bias of *Leishmania* species that causative VL and CL leishmaniasis.

**Variable**		**Meta-analysis**						
		**Pooled frequency**	**Heterogeneity**				**Publication bias**	
			***I*^**2**^**	**df**	**Cochran Q**	***P*-value**	**Egger bias**	***P*-value**
CL	*L. major*	67.3% (95% CI: 59.51–74.67%)	9625.83	134	98.6%	<0.001	−2.75	0.363
	*L. tropica*	32.1% (95% CI: 24.72–39.87%)	9693.55	134	98.6%	<0.001	3.11	0.306
VL	*L.infantum*	97.1% (95% CI: 94.6–98.8%)	39.53	21	46.9%	0.008	-0.81	0.016
	*L. tropica*	2.9% (95% CI: 1.12–5.37%)	39.53	21	46.9%	0.008	0.8175	0.016

In addition, the pooled frequency of causative agents of VL isolates was 97.1% (95% CI: 94.6–98.8%) for *L. infantum* and 2.9% (95% CI: 1.12–5.37%) for *L. tropica* (Table 3 and Supplementary Figures 3, 4). Also, other clinical forms of leishmaniasis were reported as follow: ML (*n* = 12), DCL (*n* = 5), MCL (*n* = 3), and PKDL (*n* = 2). ML cases caused by *L. major* (*n* = 7)*, L. tropica* (*n* = 2)*, L. infantum* (*n* = 2), and a mix of *L. major/L. tropica* (*n* = 1) whereas, all DCL and MCL cases caused by *L. major*, but two causative agents of PKDL were identified as *L. infantum*.

## Discussion

Leishmaniasis remains a major community health-based challenge with worldwide distribution, particularly in Iran (165). Identification of species is essential in diagnosis, treatment and epidemiological studies (165). We attempted to determine the etiological agents of human cutaneous and visceral leishmaniasis and their geographical distribution in Iran over two decades ago in the current systematic review and meta-analysis study.

According to the finding of the meta-analysis, most *Leishmania* isolates identified in Iran belonged to CL than VL cases. Every year, a large number of CL cases with a wide distribution are reported in 19 of 31 Iranian provinces, primarily in the central, southwest, east, and northeast regions. Some evidence suggests that the CL incidence rate overall has been decreasing in recent years, from 37/100,000 in 2007 to 22/100,000 in 2013. This decrease in incidence could be because appropriate public health measures such as education to residents, case finding and management, treatment, control of reservoir hosts, and distribution of repellents and nets treated with permethrin in the endemic focus of the disease have been accomplished (166).

However, at the same time, it seems like the distribution of CL has been extended to a new area (166, 167). In contrast, VL is mainly endemic in restricted regions of Iran, notably the northwest (Ardabil province) and southwest (Fars province) (7).

As illustrated by the finding of the subgroup analysis, the pool frequency of *L. major* and *L. tropica*, as causative agents of CL was 67.32% (95% CI: 59.51–74.67%) and 32.1% (95% CI: 24.72–39.87%), respectively. It can be concluded that the distribution of ZCL is higher than ACL form. According to a systematic review study conducted by Foroutan et al. rodents are the most important reservoirs of *Leishmania* species in many foci of ZCL throughout Iran (168). The most important of these rodent reservoirs are *Rhombomys opimus, Meriones libycus*, and *Nesokia indica*. The finding of this study showed that *L. major* has been reported as the predominant species of these rodents. The role of rodents in the spread of ZCL is evident (168).

The findings of this study demonstrated that the main causative agent of ZCL cases in the 14 provinces is *L. major* and the main causative agent of ACL cases in the five provinces is *L. tropica* (Figure 2). Although *L. tropica* was formerly common in many large urban areas, it has also been observed in rural areas and small cities in Iran (169). According to the findings of the two studies, four isolates of *L. turanica* were found in CL patients in the Gonbad-Kavous and Turkmen Sahara districts of Golestan province, which are the known oldest ZCL foci (90, 121). Nevertheless, it should be noted that L. major is the principal agent of ZCL in Iran. Besides, 14 isolates of L. infantum have been reported to cause CL cases (14, 22, 36, 47, 74, 122). A review of the literature showed that cases of *L. infantum* as the causative agent of CL have previously been identified in the Mediterranean (170), Southeast European countries, such as Portugal, Spain, Italy, and France (171) and the Americas (172), which is consistent with the findings.

On the other hand, the pool frequency of causative agents of VL isolates was 97.1% (95% CI: 94.6–98.8%) for *L. infantum* and 2.9% (95% CI: 1.12–5.37%) for *L. tropica*. The result of this study revealed that the main causative agent of VL in Iran is *L. infantum*. According to the results of a recent systematic review, the prevalence of HVL infection has been decreased in Iran throughout the last two decades. The maximum (3%, 95% CI: 1–5%) and minimum (0.5%, 95% CI, 0.2–0.7%) pooled prevalence of HVL was estimated in the northern and western Iranian provinces, respectively (173). It should be noted that the reason for reporting the relatively high number of cases of VL in Tehran, central Iran, as shown in Figure 3, is that these patients were referred to Tehran University Hospitals from other regions for diagnosis and treatment follow-up. Therefore, these reported cases did not belong to Tehran.

Nonetheless, despite the Iranian Center for Disease Control's (CDC) efforts to monitor and prevent HVL, new human cases of VL continue to emerge in old endemic foci. On the other hand, the disease has also emerged in new non-endemic areas of the country, such as Golestan province in north-eastern Iran (174). However, geo-climatic and environmental factors play the most important role in the emergence/reemergence of HVL in an area (174). Reasonable steps to monitor VL and prevent its spread to other areas should be taken in this respect.

Currently, molecular approaches are used for species identification, genotyping, and determine polymorphisms in *Leishmania* parasites (175). In most cases, these methods have replaced the isoenzyme method, which is the standard method for determining the species and strain of the *Leishmania* parasite (176). Molecular techniques have the potential to be more sensitive and rapid. In addition to high sensitivity and specificity, molecular methods can differentiate relapse from reinfection of disease (177).

Several DNA markers were used for DNA amplification of Leishmania spp. in the included articles, in which most kDNA and ITS1markers were used for the diagnosis of identification of species. Our finding showed that the kDNA-based PCR was the most sensitive diagnostic method for leishmaniasis and the ITS1-based PCR could be used as a sensitive/specific method to identify the Leishmania species. It is interesting to know that ITS1 is less sensitive compared to kDNA minicircles, because the copy number of rDNA (<200) is lower than the copy number of kDNA minicircles (tens of thousands). Therefore, it is more desirable to use specific primers for ITS regions and kDNA genes to diagnose the disease (103).

Phylogenetic analyses targeting the ITS1 gene are valuable and reliable tools in genetic analytical characterization of *Leishmania* parasite. This region is highly conserved among species (178). The ITS region as a target for differentiation of *Leishmania* at species and strain level has been used in different studies (102, 105, 132). As a whole, it should be noted that apply of two genetic markers simultaneously could provide more data regarding genetic map of the *Leishmania* parasite particularly in an endemic focus.

Notwithstanding that the DNA-based methods have proven to be very efficient in the identification and distinguish of *Leishmania* species, these methods also have limitations. One of these limitations is the exquisite sensitivity of these methods, and consequently false-positive PCR (179). For resolving this problem, it is necessary to use positive and negative control in each experiment simultaneously. Furthermore, preventing PCR contamination requires that this method be performed in reference laboratories. The specificity of PCR is generally controlled by several variables, including primer design, target genes, amount and purity of DNA, and type of enzyme (180).

In the end, the issue of the *Leishmania* RNA virus has become an interesting topic (181). *Leishmania* RNA virus (or LRV) is a genus of double-stranded RNA (dsRNA) virus in the family Totiviridae. LRVs exist within many species of the *Leishmania* isolates (181). Nowadays, *Leishmania* RNA virus is being extensively surveyed because it might be an important virulence factor of the infection (182). According to previous evidence, studies have been conducted to investigate the presence of *Leishmania* RNA virus in Iran. It is interesting to know that *Leishmania* RNA virus has been detected in many *L. major* species and one *L. infantum* isolated from a VL patient, and one *L. tropica* isolated from a CL patient in Iran (110, 183).

## Limitations

One of the limitations of this study was that some authors did not report isolates to belong to which province and isolates were introduced to as Iranian isolates. In addition, the limitations of the present study include: (a) use of different diagnostic techniques in the two included studies without similar results, (b) available studies with no sufficient information on identification of *Leishmania* species, and (c) variability of the sample size of the included studies. Also, people commuting between urban and rural areas has made it difficult to determine the main source of infection.

## Conclusion

Our study reconfirms that CL and VL remain important infectious diseases in Iran. In this regard, the main causative agent of ZCL and ACL in Iran is *L. major* and *L. tropica*, respectively. In addition, the findings of this study demonstrated that the main causative agent of VL in Iran is *L. infantum*. The current study provides the geographical distribution of causative species in CL and VL forms in Iran and is a source of data to help researchers and public health workers in comprehensive investigations and developing prevention programs. Based on current findings, two markers kDNA and ITS1 can be used to accurately diagnose and determine *Leishmania* species using molecular methods. Our findings highlight the need for the implementation of control measures among the patients of both CL and VL. Further attention and monitoring will be needed to improve the surveillance and effective control to reduce the incidence of leishmaniasis in Iran.

## Data Availability Statement

The original contributions presented in the study are included in the article/Supplementary Material, further inquiries can be directed to the corresponding author/s.

## Author Contributions

HH and RS conceived the presented idea and wrote the manuscript. MF and MM reviewed and commented on the findings of this work. HH, AB, and SG initially searched the literature studies and collected the data. SH analyzed and interpreted the data and methods. All authors provided critical feedback and agreed to the published version of the manuscript.

## Conflict of Interest

The authors declare that the research was conducted in the absence of any commercial or financial relationships that could be construed as a potential conflict of interest.
