# Supplementary material for: The Geographical Distribution of Human Cutaneous and Visceral Leishmania Species Identified by Molecular Methods in Iran: A Systematic Review With Meta-Analysis
Source: Front Public Health. 2021 Jun 25;9:661674. doi: 10.3389/fpubh.2021.661674 (PMC8267797; doi:10.3389/fpubh.2021.661674)
Supplement: Supplementary file 1 [file Data_Sheet_1.pdf]

Supplementary information

Supp figure 1. Forest plot graph of included studies reporting pool frequency of *L. major* in CL cases.

Supp figure 2. Forest plot graph of included studies reporting pool frequency of *L. tropica* in CL cases.

Supp figure 3. Forest plot graph of included studies reporting pool frequency of *L. infantum* in VL cases.

Supp figure 4. Forest plot graph of included studies reporting pool frequency of *L. tropica* in VL cases.

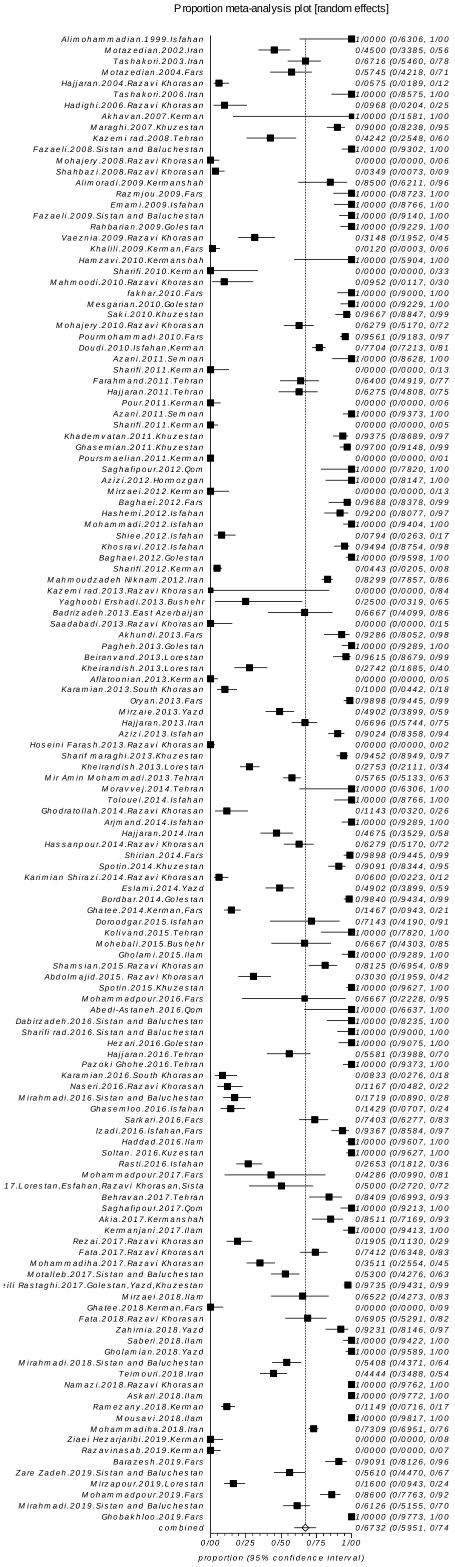
Proportion meta-analysis plot [random effects]
Alimohammadian.1999.Isfahan 1/0000 (0/6306, 1/00
Motazedian.2002.Iran 0/4500 (0/3385, 0/56
Tashakori.2003.Iran 0/6716 (0/5460, 0/78
Motazedian.2004.Fars 0/5745 (0/4218, 0/71
Hajjaran.2004.Razavi Khorasan 0/0575 (0/0189, 0/12
Tashakori.2006.Iran 1/0000 (0/8575, 1/00
Hadighi.2006.Razavi Khorasan 0/0968 (0/0204, 0/25
Akhavan.2007.Kerman 1/0000 (0/1581, 1/00
Maraghi.2007.Khuzestan 0/9000 (0/8238, 0/95
Kazemi rad.2008.Tehran 0/4242 (0/2548, 0/60
Fazaeli.2008.Sistan and Baluchestan 1/0000 (0/9302, 1/00
Mohajery.2008.Razavi Khorasan 0/0000 (0/0000, 0/06
Shahbazi.2008.Razavi Khorasan 0/0349 (0/0073, 0/09
Alimoradi.2009.Kermanshah 0/8500 (0/6211, 0/96
Razmjou.2009.Fars 1/0000 (0/8723, 1/00
Emami.2009.Isfahan 1/0000 (0/8766, 1/00
Fazaeli.2009.Sistan and Baluchestan 1/0000 (0/9140, 1/00
Rahbarian.2009.Golestan 1/0000 (0/9229, 1/00
Vaeznia.2009.Razavi Khorasan 0/3148 (0/1952, 0/45
Khalili.2009.Kerman,Fars 0/0120 (0/0003, 0/06
Hamzavi.2010.Kermanshah 1/0000 (0/5904, 1/00
Sharifi.2010.Kerman 0/0000 (0/0000, 0/33
Mahmoodi.2010.Razavi Khorasan 0/0952 (0/0117, 0/30
fakhar.2010.Fars 1/0000 (0/9000, 1/00
Mesgarian.2010.Golestan 1/0000 (0/9229, 1/00
Saki.2010.Khuzestan 0/9667 (0/8847, 0/99
Mohajery.2010.Razavi Khorasan 0/6279 (0/5170, 0/72
Pourmohammadi.2010.Fars 0/9561 (0/9183, 0/97
Doudi.2010.Isfahan,Kerman 0/7704 (0/7213, 0/81
Azani.2011.Semnan 1/0000 (0/8628, 1/00
Sharifi.2011.Kerman 0/0000 (0/0000, 0/13
Farahmand.2011.Tehran 0/6400 (0/4919, 0/77
Hajjaran.2011.Tehran 0/6275 (0/4808, 0/75
Pour.2011.Kerman 0/0000 (0/0000, 0/06
Azani.2011.Semnan 1/0000 (0/9373, 1/00
Sharifi.2011.Kerman 0/0000 (0/0000, 0/05
Khademvatan.2011.Khuzestan 0/9375 (0/8689, 0/97
Ghasemian.2011.Khuzestan 0/9700 (0/9148, 0/99
Poursmaelian.2011.Kerman 0/0000 (0/0000, 0/01
Saghafipour.2012.Qom 1/0000 (0/7820, 1/00
Azizi.2012.Hormozgan 1/0000 (0/8147, 1/00
Mirzaei.2012.Kerman 0/0000 (0/0000, 0/13
Baghaei.2012.Fars 0/9688 (0/8378, 0/99
Hashemi.2012.Isfahan 0/9200 (0/8077, 0/97
Mohammadi.2012.Isfahan 1/0000 (0/9404, 1/00
Shiee.2012.Isfahan 0/0794 (0/0263, 0/17
Khosravi.2012.Isfahan 0/9494 (0/8754, 0/98
Baghaei.2012.Golestan 1/0000 (0/9598, 1/00
Sharifi.2012.Kerman 0/0443 (0/0205, 0/08
Mahmoudzadeh Niknam.2012.Iran 0/8299 (0/7857, 0/86
Kazemi rad.2013.Razavi Khorasan 0/0000 (0/0000, 0/84
Yaghoobi Ershadi.2013.Bushehr 0/2500 (0/0319, 0/65
Badrizadeh.2013.East Azerbaijan 0/6667 (0/4099, 0/86
Saadabadi.2013.Razavi Khorasan 0/0000 (0/0000, 0/15
Akhundi.2013.Fars 0/9286 (0/8052, 0/98
Pagheh.2013.Golestan 1/0000 (0/9289, 1/00
Beiranvand.2013.Lorestan 0/9615 (0/8679, 0/99
Kheirandish.2013.Lorestan 0/2742 (0/1685, 0/40
Aflatoonian.2013.Kerman 0/0000 (0/0000, 0/05
Karamian.2013.South Khorasan 0/1000 (0/0442, 0/18
Oryan.2013.Fars 0/9898 (0/9445, 0/99
Mirzaie.2013.Yazd 0/4902 (0/3899, 0/59
Hajjaran.2013.Iran 0/6696 (0/5744, 0/75
Azizi.2013.Isfahan 0/9024 (0/8358, 0/94
Hoseini Farash.2013.Razavi Khorasan 0/0000 (0/0000, 0/02
Sharif maraghi.2013.Khuzestan 0/9452 (0/8949, 0/97
Kheirandish.2013.Lorestan 0/2753 (0/2111, 0/34
Mir Amin Mohammadi.2013.Tehran 0/5765 (0/5133, 0/63
Moravvej.2014.Tehran 1/0000 (0/6306, 1/00
Tolouei.2014.Isfahan 1/0000 (0/8766, 1/00
Ghodratollah.2014.Razavi Khorasan 0/1143 (0/0320, 0/26
Arjmand.2014.Isfahan 1/0000 (0/9289, 1/00
Hajjaran.2014.Iran 0/4675 (0/3529, 0/58
Hassanpour.2014.Razavi Khorasan 0/6279 (0/5170, 0/72
Shirian.2014.Fars 0/9898 (0/9445, 0/99
Spotin.2014.Khuzestan 0/9091 (0/8344, 0/95
Karimian Shirazi.2014.Razavi Khorasan 0/0600 (0/0223, 0/12
Eslami.2014.Yazd 0/4902 (0/3899, 0/59
Bordbar.2014.Golestan 0/9840 (0/9434, 0/99
Ghatee.2014.Kerman,Fars 0/1467 (0/0943, 0/21
Doroodgar.2015.Isfahan 0/7143 (0/4190, 0/91
Kolivand.2015.Tehran 1/0000 (0/7820, 1/00
Mohebali.2015.Bushehr 0/6667 (0/4303, 0/85
Gholami.2015.Ilam 1/0000 (0/9289, 1/00
Shamsian.2015.Razavi Khorasan 0/8125 (0/6954, 0/89
Abdolmajid.2015. Razavi Khorasan 0/3030 (0/1959, 0/42
Spotin.2015.Khuzestan 1/0000 (0/9627, 1/00
Mohammadpour.2016.Fars 0/6667 (0/2228, 0/95
Abedi-Astaneh.2016.Qom 1/0000 (0/6637, 1/00
Dabirzadeh.2016.Sistan and Baluchestan 1/0000 (0/8235, 1/00
Sharifi rad.2016.Sistan and Baluchestan 1/0000 (0/9000, 1/00
Hezari.2016.Golestan 1/0000 (0/9075, 1/00
Hajjaran.2016.Tehran 0/5581 (0/3988, 0/70
Pazoki Ghohe.2016.Tehran 1/0000 (0/9373, 1/00
Karamian.2016.South Khorasan 0/0833 (0/0276, 0/18
Naseri.2016.Razavi Khorasan 0/1167 (0/0482, 0/22
Mirahmadi.2016.Sistan and Baluchestan 0/1719 (0/0890, 0/28
Ghasemloo.2016.Isfahan 0/1429 (0/0707, 0/24
Sarkari.2016.Fars 0/7403 (0/6277, 0/83
Izadi.2016.Isfahan,Fars 0/9367 (0/8584, 0/97
Haddad.2016.Ilam 1/0000 (0/9607, 1/00
Soltan. 2016.Kuzestan 1/0000 (0/9627, 1/00
Rasti.2016.Isfahan 0/2653 (0/1812, 0/36
Mohammadpour.2017.Fars 0/4286 (0/0990, 0/81
17.Lorestan,Esfahan,Razavi Khorasan,Sista 0/5000 (0/2720, 0/72
Behravan.2017.Tehran 0/8409 (0/6993, 0/93
Saghafipour.2017.Qom 1/0000 (0/9213, 1/00
Akia.2017.Kermanshah 0/8511 (0/7169, 0/93
Kermanjani.2017.Ilam 1/0000 (0/9413, 1/00
Rezai.2017.Razavi Khorasan 0/1905 (0/1130, 0/29
Fata.2017.Razavi Khorasan 0/7412 (0/6348, 0/83
Mohammadiha.2017.Razavi Khorasan 0/3511 (0/2554, 0/45
Motalleb.2017.Sistan and Baluchestan 0/5300 (0/4276, 0/63
ili Rastaghi.2017.Golestan,Yazd,Khuzestan 0/9735 (0/9431, 0/99
Mirzaei.2018.Ilam 0/6522 (0/4273, 0/83
Ghatee.2018.Kerman,Fars 0/0000 (0/0000, 0/09
Fata.2018.Razavi Khorasan 0/6905 (0/5291, 0/82
Zahirnia.2018.Yazd 0/9231 (0/8146, 0/97
Saberi.2018.Ilam 1/0000 (0/9422, 1/00
Gholamian.2018.Yazd 1/0000 (0/9589, 1/00
Mirahmadi.2018.Sistan and Baluchestan 0/5408 (0/4371, 0/64
Teimouri.2018.Iran 0/4444 (0/3488, 0/54
Namazi.2018.Razavi Khorasan 1/0000 (0/9762, 1/00
Askari.2018.Ilam 1/0000 (0/9772, 1/00
Ramezany.2018.Kerman 0/1149 (0/0716, 0/17
Mousavi.2018.Ilam 1/0000 (0/9817, 1/00
Mohammadiha.2018.Iran 0/7309 (0/6951, 0/76
Ziaei Hezarjaribi.2019.Kerman 0/0000 (0/0000, 0/08
Razavinasab.2019.Kerman 0/0000 (0/0000, 0/07
Barazesh.2019.Fars 0/9091 (0/8126, 0/96
Zare Zadeh.2019.Sistan and Baluchestan 0/5610 (0/4470, 0/67
Mirzapour.2019.Lorestan 0/1600 (0/0943, 0/24
Mohammadpour.2019.Fars 0/8600 (0/7763, 0/92
Mirahmadi.2019.Sistan and Baluchestan 0/6126 (0/5155, 0/70
Ghobakhloo.2019.Fars 1/0000 (0/9773, 1/00
combined 0/6732 (0/5951, 0/74
0/00
0/25
0/50
0/75
1/00
proportion (95% confidence interval)

Supp figure 1. Forest plot graph of included studies reporting pool frequency of *L. major* in CL cases.

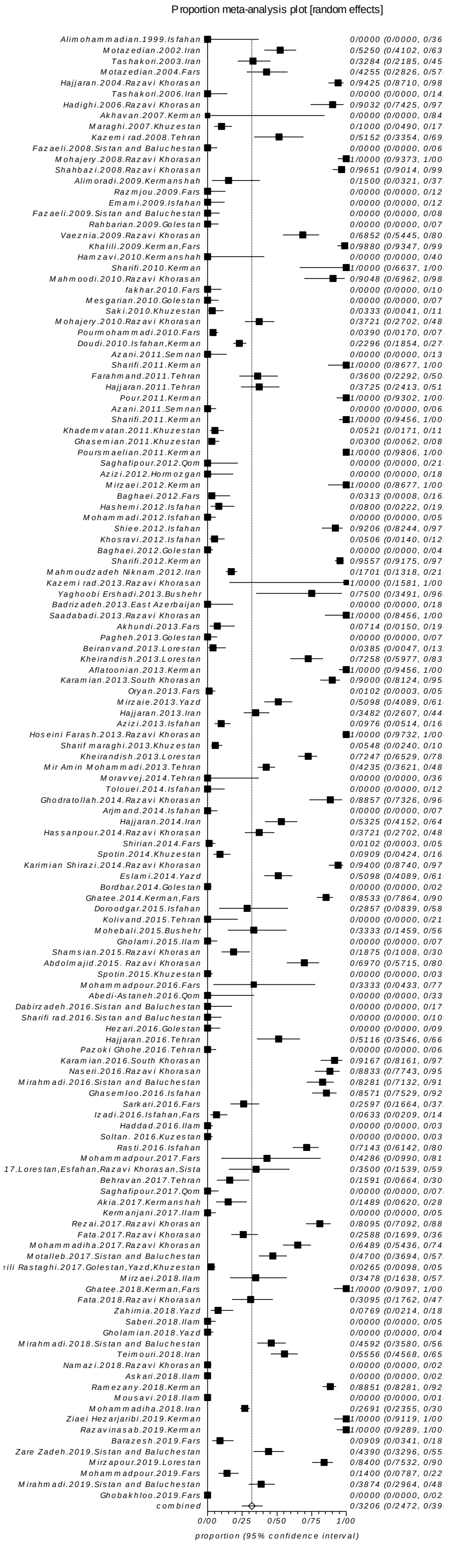

Supp figure2. Forest plot graph of included studies reporting pool frequency of *L. tropica* in CL cases.

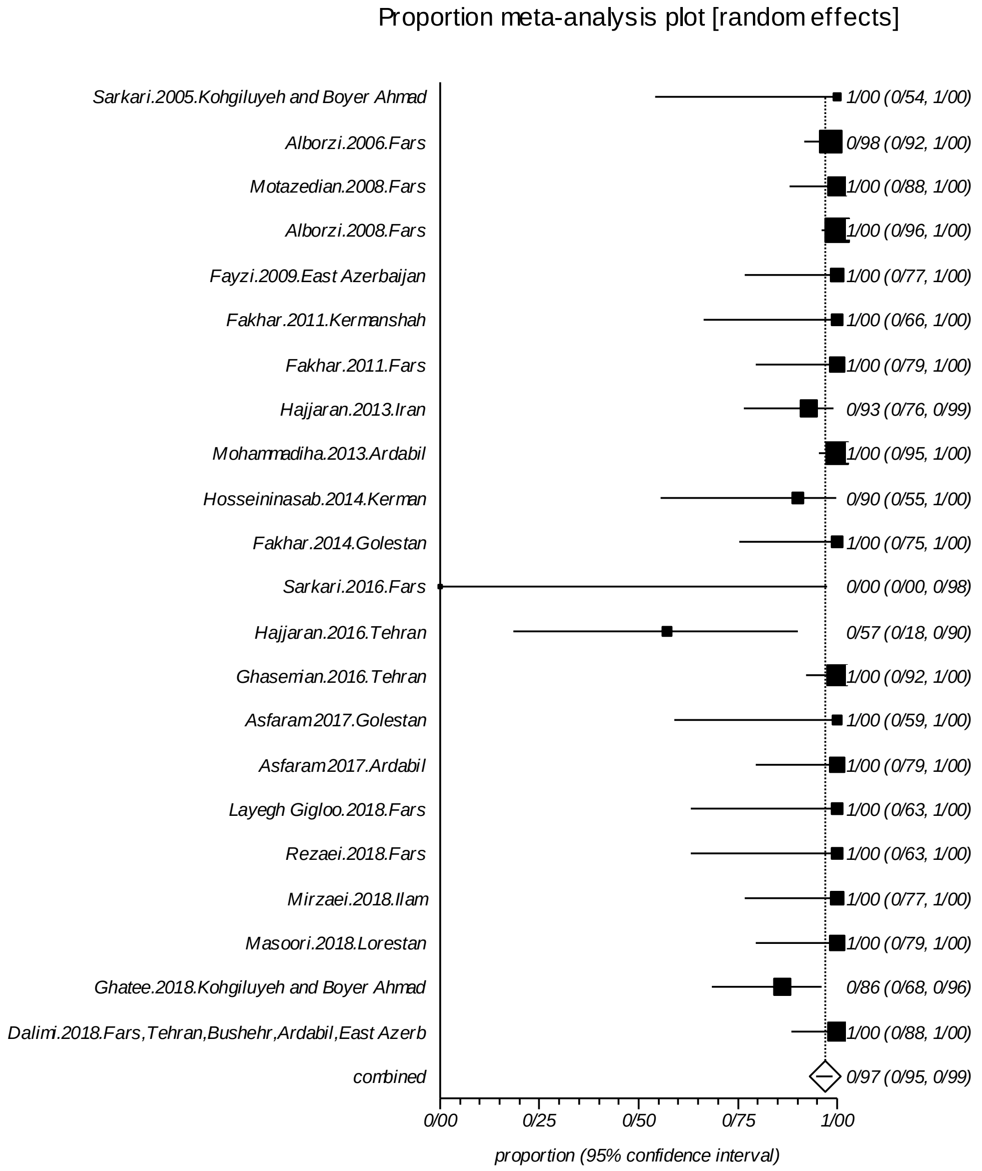

Supp figure 3. Forest plot graph of included studies reporting pool frequency of *L. infantum* in VL cases.

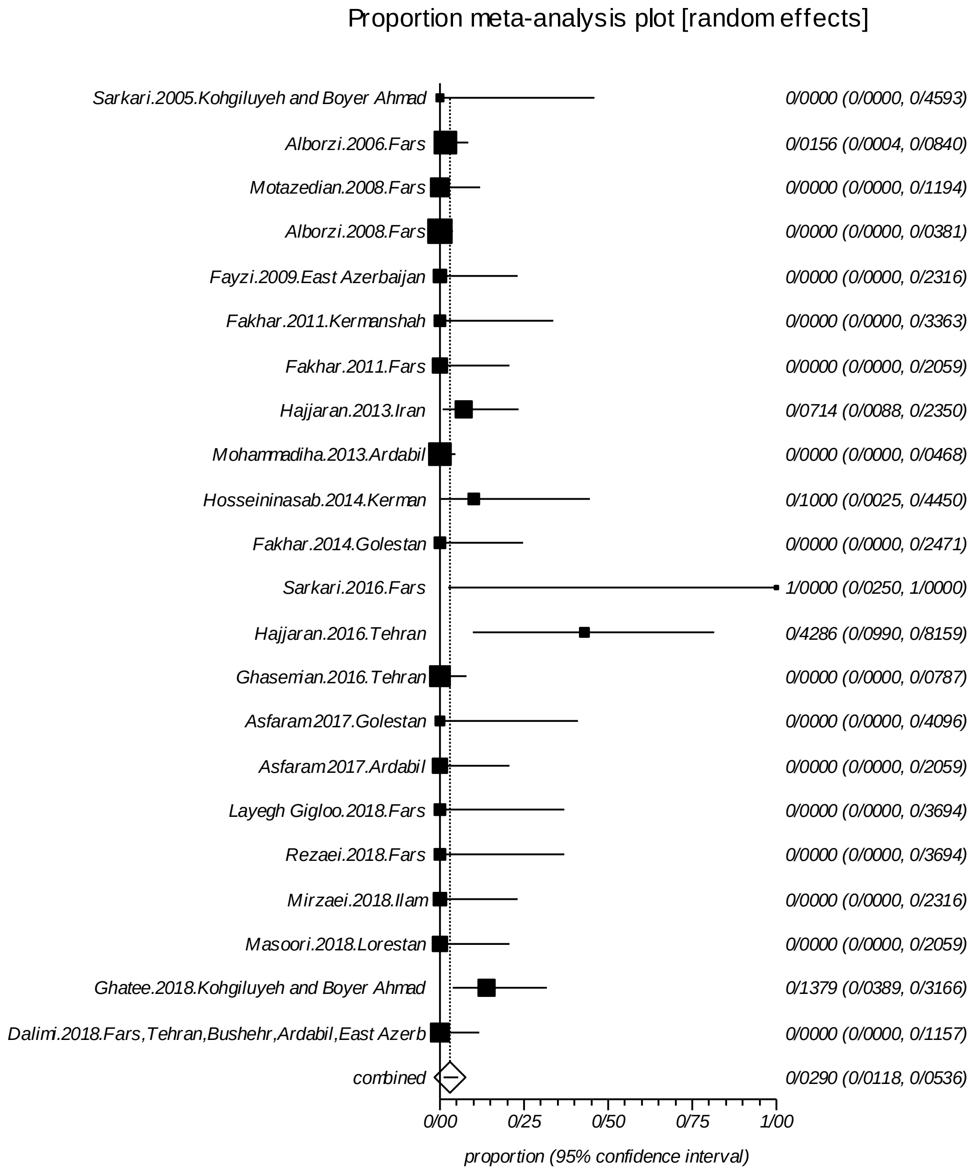

Supp figure 4. Forest plot graph of included studies reporting pool frequency of *L. tropica* in VL cases.
